# Shikonin inhibits multiple tumor malignant phenotypes and is associated with Hedgehog pathway downregulation in lung adenocarcinoma

**DOI:** 10.1038/s41598-025-28080-9

**Published:** 2025-12-24

**Authors:** Hong-Gang Wang, Ming-Xia Lu, Mei-Ling Sheng, Ya-Bo Lou, Yuan-Chao Xiao, An Guo, Zheng-Hong Yu

**Affiliations:** 1 The First Clinical Medical College of Nanjing University of Chinese Medicine, Nanjing, 210004 Jiangsu China; 2 The Affiliated Jinhua Hospital of Wenzhou Medical University, Wenzhou, 321000 Zhejiang China; 3 Department of Hyperbaric Oxygen Treatment Center, Jinhua People’s Hospital, Jinhua, 321000 Zhejiang China; 4 Department of Respiratory and Critical Care Medicine, Jinhua People’s Hospital, Jinhua, 321000 Zhejiang China; 5 Jinling Hospital, Affiliated Hospital of Medical School, Nanjing University, Nanjing, 210016 Jiangsu China

**Keywords:** Shikonin, Non-small-cell lung cancer, Lung adenocarcinoma, Hedgehog signaling pathway, Cancer, Cell biology, Oncology

## Abstract

Shikonin (SH) is a natural component extracted from *Lithospermum erythrorhizon* Siebold & Zucc, a traditional Chinese medicine, which has been shown to have anti-tumor effects in recent years. However, the specific mechanism behind these effects has not yet been reported. Thus, it is important to clarify the specific anti-tumor mechanism of SH and then develop it as a new anti-tumor drug. The objective of this study was to investigate the anti-tumor effect of SH on lung adenocarcinoma and its associated mechanism. SH was co-cultured with A549 cells, and the effects of SH on tumor-related phenotypes were evaluated via methods such as CCK-8, transwell, and TUNEL assays. Transcriptomics was performed to investigate the expression profile of SH-regulated genes as well as the signaling pathways involved. Moreover, the anti-tumor effect of SH was verified using a mouse lung cancer xenograft model. SH inhibited the proliferation, metastasis, and invasion of A549 cells, promoted apoptosis, and regulated the expression of malignant phenotype-related markers. The transcriptomic analysis indicated that SH regulated the expression of various malignant phenotype-related mRNAs and inhibited the Hedgehog pathway activation. Furthermore, SH improved the general state of lung cancer-bearing mice, suppressed the growth of transplanted tumors, and inhibited the expression of tumor markers. The findings of this study are the first to reveal the anti-lung adenocarcinoma effect of SH from multiple perspectives—namely, increasing cancer cell apoptosis and suppressing cancer cell proliferation, metastasis, and invasion by inhibiting the Hedgehog pathway activation.

**Supplementary Information:**

The online version contains supplementary material available at 10.1038/s41598-025-28080-9.

## Introduction

Non-small cell lung cancer (NSCLC) is a type of lung malignancy that originates from the bronchial mucosa, glandular tissue, and alveolar epithelium, accounting for 85% to 90% of all lung cancers. Its morbidity and mortality rates rank first among malignant tumors, and lung adenocarcinoma is the most common subtype of NSCLC^1^. Although patients with NSCLC or lung adenocarcinoma in stages I and II can receive good clinical benefits from timely treatment, most patients are diagnosed at more advanced stages (IIIA/B). Several real-world studies have shown that the 5-year overall survival (OS) for lung adenocarcinoma patients is consistently less than 20%^2^. Moreover, the side effects and immune-related adverse events (irAE) associated with radiotherapy, chemotherapy, and immunotherapy pose great clinical challenges. Thus, there exists an urgent need to identify potent drugs with limited side effects for the management of lung adenocarcinoma.

It is well known that natural plant-derived drugs have multi-level, multi-target, and collaborative intervention effects. They are also associated with fewer side effects. Shikonin (SH), a naphthoquinone compound, represents a major active component of *Lithospermum erythrorhizon* Siebold & Zucc, a traditional Chinese medicine^3^. Shikonin has shown some inhibitory effects on tumor growth in studies of small cell lung cancer, liver cancer, and cervical cancer. However, there are currently no reports on the anti-lung adenocarcinoma effects of shikonin and its mechanism. Therefore, systematic research on these issues is crucial.

This study evaluated the therapeutic effects of SH on lung adenocarcinoma and its ability to ameliorate its malignant phenotype using human lung adenocarcinoma A549 cells. Transcriptomics was used to explore potential targets and signaling pathways for SH. Finally, the underlying mechanisms were validated using a xenograft model. These findings reveal potential mechanisms by which SH can treat lung adenocarcinoma, laying a solid foundation for its drug development and future clinical application.

## Materials and methods

### Chemicals and reagents

The human NSCLC A549 cell line was from the Shanghai Cell Bank of the Chinese Academy of Science. Lewis cell line for mouse lung cancer was purchased from Wuhan Pronosei Life Science Technology Co., Ltd. The SH utilized in the present study was provided by Baoji Herbest Bio-Tech Co., Ltd. located in Baoji, China. The DMSO and tretinoin were supplied by Beijing Solarbio Science & Technology Co., Ltd. (Beijing, China). Sodium pentobarbital is from Shanghai Fluoride Chemical Co., Ltd. The CCK-8, EdU, TUNEL, and transwell assay kits were supplied by Nanjing Jiancheng Bioengineering Institute located in Nanjing, China. The poly(ADP-ribose) polymerase 1 (PARP1), Ki-67, matrix metalloproteinase 7 (MMP-7), Snail, hypoxia-inducible factor 1α (HIF-1α), B-cell lymphoma 2 (Bcl-2), Bcl-2-associated X protein (BAX), apoptosis-related factor (Fas), Fas ligand (FasL), mini-chromosome maintenance protein 2 (MCM2), cyclin D, p21, vascular endothelial growth factor (VEGF), E-cadherin (E-cad), N-cadherin (N-cad), AMDM, and epidermal growth factor (EGF) antibodies were all supplied by Proteintech Group, Inc (Wuhan, China). The purmorphamine (PM) was supplied by Yeasen Biotech Co., Ltd. located in Shanghai, China. Finally, the TRIzol and WB chemiluminescence reagents were purchased from Ambion Thermo Fisher Scientific CN (Shanghai, China).

### Cell lines

The human NSCLC A549 cell line utilized in the present study was supplied by the Shanghai Cell Bank of the Chinese Academy of Science (Shanghai, China). The A549 cells were grown in DMEM medium containing 10% FBS and 100 U/mL penicillin–streptomycin at 37 °C and 5% CO_2_ passaged every 3 days. When the cells grew well and reached the logarithmic growth phase, they were digested using trypsin, centrifuged, and resuspended in a medium to obtain a single-cell suspension, and then the cells were subjected to subsequent experiments according to experimental requirements.

### CCK-8

The A549 cells in the logarithmic growth phase and in good growth status were seeded at 5 × 10^3^ cells/well in 96-well plates and subsequently cultured at 37 °C and 5% CO_2_ overnight. SH was added to the wells at concentrations of 0, 2.5, 5, 7.5, 10, 12.5, 15, 17.5, and 20 μmol/L, and the cells were incubated for 24 h. Subsequently, the CCK-8 reagent was given to every well at a volume of 10 μL per well, which was followed by cell incubation at 37 °C for 4 h. The absorbance value of every well was determined at a wavelength of 450 nm using a microplate reader. To investigate the time-dependent effects of SH on cell viability, the A549 cells were stimulated with SH at concentrations of 10, 15, and 20 μmol/L for 6, 12, 18, 24, 36, and 48 h. Subsequently, the cells underwent incubation with 10 μL of CCK-8 reagent at a temperature of 37 °C for a period of 4 h. Using a microplate reader, the absorbance of each well was measured at a wavelength of 450 nm.

### EdU labeling

After completion of the EdU labeling experiments, the culture medium was removed. The cells were then fixed using 1 mL of 4% paraformaldehyde at room temperature for 15 min. This was followed by incubation with 1 mL of PBS with 0.3% Triton X-100 per well at room temperature for 15 min. After 0.5 mL of the click reaction solution was added to every well. The cells were then cultured under darkness at room temperature for 30 min before removal of the click reaction solution and washing. Subsequently, the cells were incubated with the Hoechst 33342 staining solution at a dilution of 1:1000 in PBS in the dark at room temperature for 10 min before the addition of a mounting solution with an anti-fluorescence quencher to seal the wells. Finally, observe with a fluorescence microscope.

### TUNEL assay

The slides with adherent cells were immersed in 4% paraformaldehyde and fixed at room temperature for 15 min. Proteinase K solution (2 mg/mL) was diluted to 20 μg/mL using deionized water at a 1:100 ratio. Next, 100 μL of the diluted proteinase K solution was added to each slide, ensuring complete coverage, and the slides were then placed at room temperature for 20 min. Subsequently, 5 × Equilibration Buffer was prepared by diluting it with deionized water at a ratio of 1:5. Afterward, each slide was covered completely with 100 μL of 1 × Equilibration Buffer and kept at room temperature for 15 min. BrightRed Labeling Mix was thawed on ice, and the TdT incubation buffer was made up as per the manufacturer’s instructions. Following the addition of TdT incubation buffer, the samples were incubated at 37 °C for 60 min in a humidified chamber. Next, the slides were incubated with DAPI for 5 min under darkness to stain the nuclei, followed by any excess DAPI being washed away with PBST. The slides were sealed with a mounting solution with an anti-fluorescence quencher. Lastly, the specimens were examined, and the required figures were taken with the aid of a fluorescence microscope.

### Transwell assay

In terms of cell migration, single-cell suspensions of various cell groups were prepared in a serum-free culture medium and seeded at a density of 1 × 10^5^ cells per mL in the upper chamber of the Transwell. SH was added in the previously specified concentration gradients. The lower 24-well plate contained a culture medium with 20% FBS. Following the time-gradient incubation at 37 °C, any non-migratory cells in the upper chamber were discarded. The cells on the lower chamber membrane were then fixed using paraformaldehyde, stained with crystal violet staining solution, and examined, photographed, and counted under an inverted microscope.

With regard to cell invasion, matrigel was dissolved in the RPMI-1640 medium, which was evenly spread in the Transwell chambers. The Transwell chambers were then kept overnight in a refrigerator at 4 °C. SH was added in the cells in the previously specified concentration gradients. Next, the single-cell suspensions of 1 × 10^5^ cells/mL were seeded in the upper chamber. The lower chamber was filled with a culture medium with 20% FBS. Following incubation at 37 °C and 5% CO_2_ in the previously specified time gradient, the cells in the chambers were washed, fixed with methanol, stained using crystal violet, observed, photographed, and counted under a microscope.

### Cell scratch assay

Healthy growing A549 cells (density: 5 × 10^4^ cells/mL) were evenly distributed on the six-well plates and incubated at 37 °C and 5% CO_2_. Vertical scratch lines were created using a 10 μL pipette tip when the cells reached around 90% confluency. Any cell debris was washed away using PBS. Different concentrations of SH (10 μL) were then added. The plates were photographed under a microscope for the purpose of labeling, which was marked as 0 h. The cells were incubated at 37 °C and 5% CO_2_ for 24 h, followed by observation and photography of the cell movement under an inverted microscope.

### Immunofluorescence

The slides with adherent cells were rinsed with PBS in a culture plate and fixed with 4% paraformaldehyde. Permeabilization was performed at room temperature using 0.5% Triton X-100 (made up in PBS) for 20 min. Subsequently, blocking was performed using goat serum at room temperature for 30 min. The cells were then incubated with the primary antibodies overnight at 4 °C. Next, the cells were incubated with the fluorescent secondary antibodies at 37 °C for 60 min in a humid chamber. Following this, the cells were incubated with DAPI for 5 min under dark conditions, and any excess DAPI was removed. The slides were sealed using a mounting medium with an anti-fluorescence quencher. Lastly, the slides were examined and the required images were captured with the aid of a fluorescence microscope.

### RNA-seq and analysis

The precipitated A549 cells provided RNA samples that were delivered to Guangzhou GeneDenovo Biotechnology Co., Ltd. (Guangzhou, China) to construct the RNA-seq libraries. The total RNA was isolated, and the mRNA was enriched for cDNA synthesis and sequencing. Transcriptome RNA sequencing was conducted using the Illumina NovaSeq 6000 platform, which generated double-ended reads of 150 bp (PE150). Identification of differentially expressed genes (DEGs) was performed with edgeR (|logFC|> 1 and FDR < 0.05). The DEGs were then subjected to the Kyoto Encyclopedia of Genes and Genomes (KEGG) (https://www.kegg.jp/) pathway enrichment analysis and gene set enrichment analysis (GSEA).

### Construction of tumor-bearing mice and sampling

This study was conducted in accordance with the ARRIVE 2.0 guidelines^4^. All animal procedures were reviewed and approved by the Ethics Committee for Laboratory Animal Welfare of Shaanxi University of Chinese Medicine (Approval code: SUCMDL20211010023) and complied with national and institutional regulations on laboratory animal welfare and ethics in China. All procedures were performed by trained personnel with measures in place to minimize pain and distress. The C57BL/6 mice (n = 60) were housed in a specific-pathogen-free area at the Experimental Animal Center of Shaanxi University of Chinese Medicine. They were then randomly divided into groups: Model group; SH high-, medium-, and low-dose (SH-H, -M, and -L) groups; and cyclophosphamide (CTX) group. Each group contained 12 mice. Mice were anesthetized via intraperitoneal injection of 1% sodium pentobarbital (40 mg/kg). Lewis cells (2 × 10^6^) were then mixed at a 1:1 (v/v) ratio with 0.2 mL physiological (normal) saline and injected into the axillary region of each mouse. One week later, the treatment groups received intraperitoneal injections of the corresponding drugs: the SH high-, medium-, and low-dose groups received 4, 2, and 1 mg/kg of SH, respectively; the CTX group received a 50 mg/kg dose of CTX; and the Model group received the same volume of saline. All the mice were treated every 3 days for 15 days. After the final dose administration, 1% pentobarbital sodium (200 mg/kg, intraperitoneal injection) was used for euthanasia. The tumor tissues were photographed, weighed, and then embedded in paraffin for the subsequent experiments.

### Immunohistochemistry

The tumor tissue sections were deparaffinized with xylene and gradient ethanol, which was followed by antigen retrieval in 95 °C EDTA buffer. After blocking with endogenous peroxidase and washing tissue sections, the cells were incubated at room temperature with 5% goat serum for 30 min, and they were then treated with Ki-67, VEGFA, and CD31 antibodies overnight at 4 °C. Following washing, the tissue sections were treated with HRP-conjugated secondary antibodies at room temperature for 30 min. The cells were visualized with DAB, followed by hematoxylin counterstaining to stain nuclei. Lastly, the specimens were examined and the required figures were captured under a light microscope.

### Western blot (WB)

The cells were lysed using RIPA buffer. The protein concentrations were detected via a BCA protein assay. The protein samples were subjected to denaturation in an SDS loading buffer at 95 °C for 5 min. The denatured protein samples were separated by means of sodium dodecyl-sulfate polyacrylamide gel electrophoresis (SDS-PAGE) and electrotransferred onto PVDF membranes. Blocking was performed by using 5% non-fat milk for 1 min. The samples were treated with primary antibodies overnight at 4 °C, followed by secondary antibodies at room temperature for 1 min. Chemiluminescent reagents (Millipore) were used for the signal development, while an imaging system (Bio-Rad) was used for detection.

### Reverse transcription (RT)-PCR

The total RNA was prepared using an RNA extraction kit (Beijing Solarbio Science & Technology Co., Ltd., Beijing, China). Subsequently, the RNA was reverse transcribed into cDNA using a HiScript II 1st Strand cDNA Synthesis Kit (Vazyme). The qPCR was then performed with the use of ChamQ SYBR Color qPCR Master Mix (Vazyme) and a Bio-Rad CFX96 detection system. The relative fold change in the gene expression was calculated by comparing the 2^−ΔΔCt^ values, using β-actin serving as the internal reference. The sequences of the primers are listed in Supplementary Table 1.

### Statistical analysis

The paper presents quantitative data as mean ± standard deviation. Statistical analyses were carried out with the use of SPSS 22.0 software (IBM). Comparisons between groups were made using one-way analysis of variance (ANOVA), and multiple comparisons were made using the least significant difference (LSD). Statistical significance was set at *p* < 0.05, *p* < 0.01, or *p* < 0.001.

## Results

### SH inhibits proliferation, migration, and invasion and promotes apoptosis in A549 cells

**Fig. 1:**
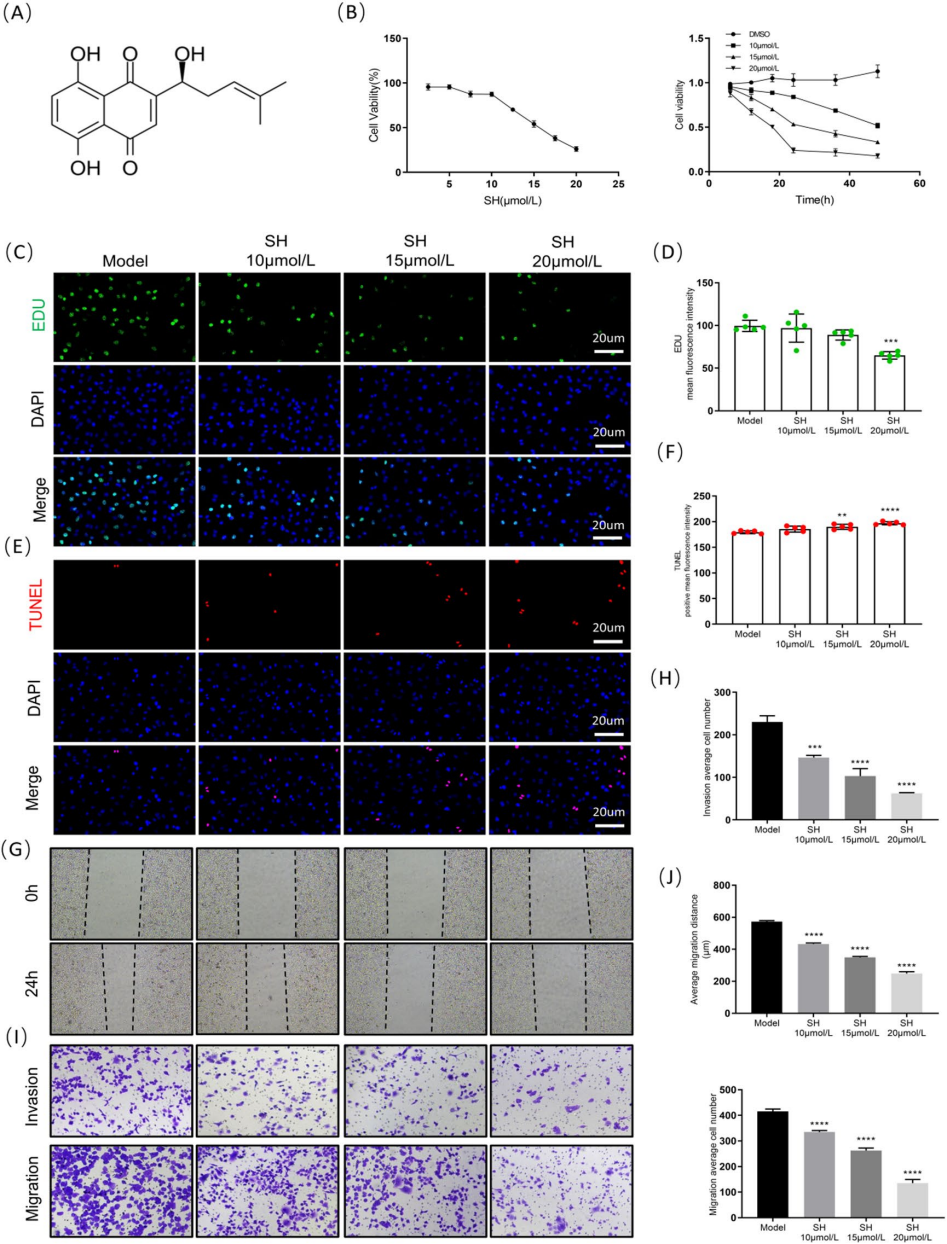
SH inhibits A549 cell proliferation, migration, and invasion and promotes apoptosis (mean ± SD, n = 5 (independent cell cultures)). (**A**) Chemical structure of Shikonin. (**B**) Cell viability. (**C**) Representation images of EDU staining (scale bar: 20 μm, × 400). (**D**) Measuring the intensity of positive EDU staining. (**E**) TUNEL staining of tumor cells (scale bar: 20 μm, × 400). (**F**) Proportion of cells exhibiting positive TUNEL staining. (**G**) Representative images of Cell scratch assay at 0 and 24 h. (**H**) Invasion average cell number. (**I**) Transwell and invasion assay. (**J**) The number of migration cells were counted in A549 cell. **p < 0.01; ***p < 0.001 and ****p < 0.0001, compared with Model group, one way ANOVA.

The structure of SH, a naphthoquinone compound extracted from *Lithospermum erythrorhizon* Siebold & Zucc root, is depicted in Fig. 1A. A549 cells were co-cultured with SH in gradients of 2.5–20 μmol/L for 24 h, showing a dose-dependent decrease in cell viability according to the CCK-8 results. We selected 10, 15, and 20 μmol/L concentrations for the subsequent time gradient experiment. Cell viability was further inhibited with a prolonged intervention time between 6 and 48 h (Fig. 1B). Based on the experimental results, we selected 24 h as the subsequent intervention time. EdU and TUNEL staining allowed us to further evaluate the cell proliferation and apoptosis, demonstrating that SH significantly inhibited cell proliferation and promoted apoptosis (Fig. 1C–F). Subsequently, the scratch and transwell assays assessed the tumor cell migration and invasion, respectively. SH significantly inhibited the A549 cells’ migration distance and invasion quantity (Fig. 1G–J). In summary, SH can effectively inhibit cell proliferation, migration, and invasion and promote cell apoptosis in A549 cells.

### Transcriptomic analysis of A549 cells treated with SH

**Fig. 2:**
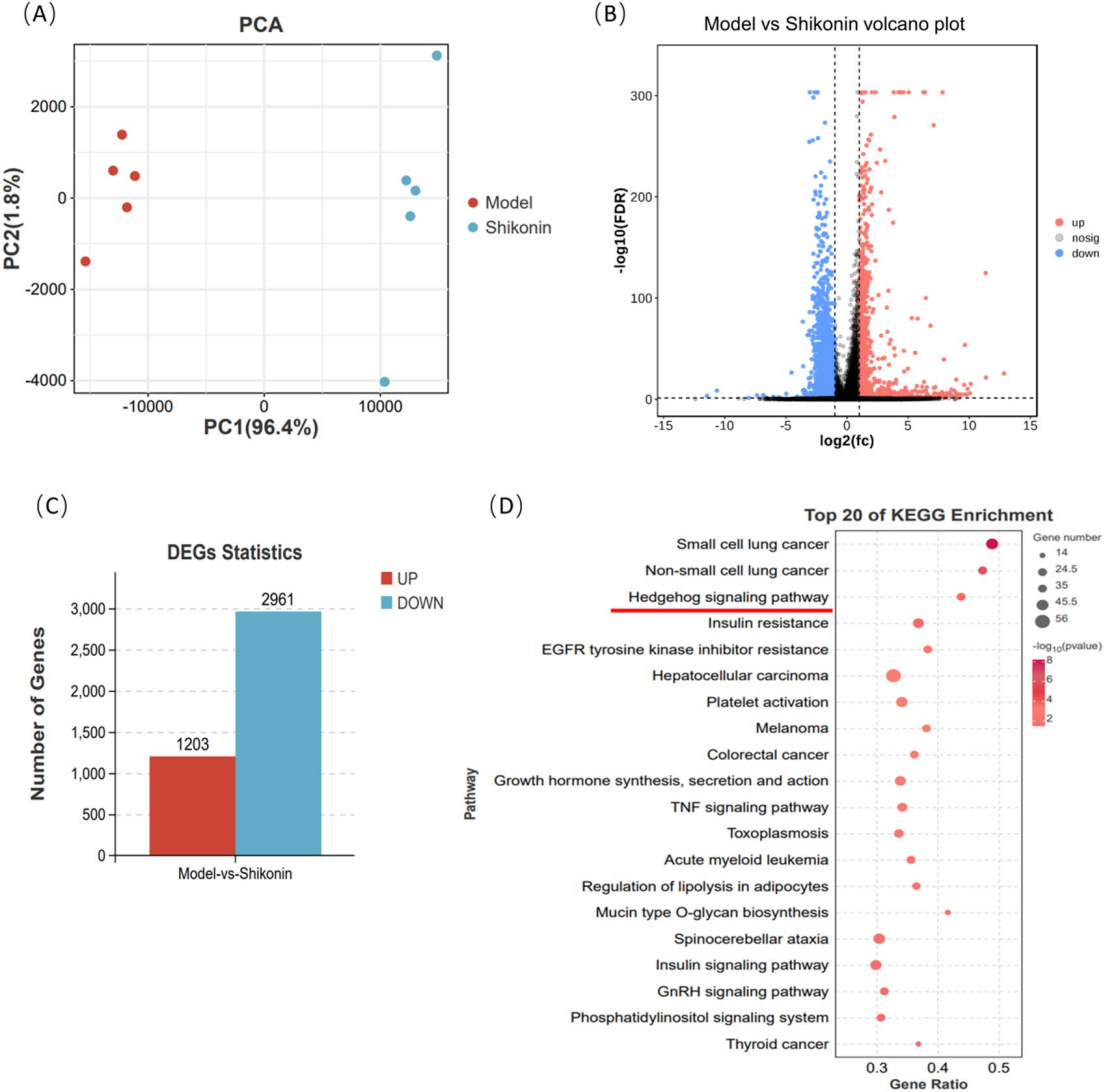
Transcriptomic analysis of A549 cells treated with SH (n = 5 (independent cell cultures)). (**A**) PCA scatter plot illustrating transcriptome variations between the Model and Shikonin groups. (**B**) Volcano plot showing differentially expressed genes (DEGs) between Model versus Shikonin. (**C**) Quantities of differentially expressed genes, where up-regulated genes are indicated in red and down-regulated genes are shown in blue. (**D**) Top 20 KEGG pathway enrichments for differentially expressed genes (Model vs. Shikonin).

To systematically analyze the effect of SH on the lung adenocarcinoma malignant phenotypes, we performed a transcriptomic analysis. Our principal component analysis (PCA) identified two distinct clusters between the Model and SH groups (Fig. 2A). The DEGs were screened out with a threshold of *p* < 0.05 and log2 FC > 1.5. When compared with the Model group, the SH-H group had 4164 DEGs in total, of which 1203 genes were upregulated and 2961 genes were downregulated (Fig. 2B). Subsequently, we performed a KEGG enrichment analysis to analyze and predict the functions of these DEGs. The top 20 pathways were primarily “Small cell lung cancer”, “Non-small cell lung cancer”, “Hedgehog signaling pathway”, and “Insulin resistance” (Fig. 2C).

### SH regulates the expression of DEGs related to tumor malignant phenotypes in A549 cells

**Fig. 3:**
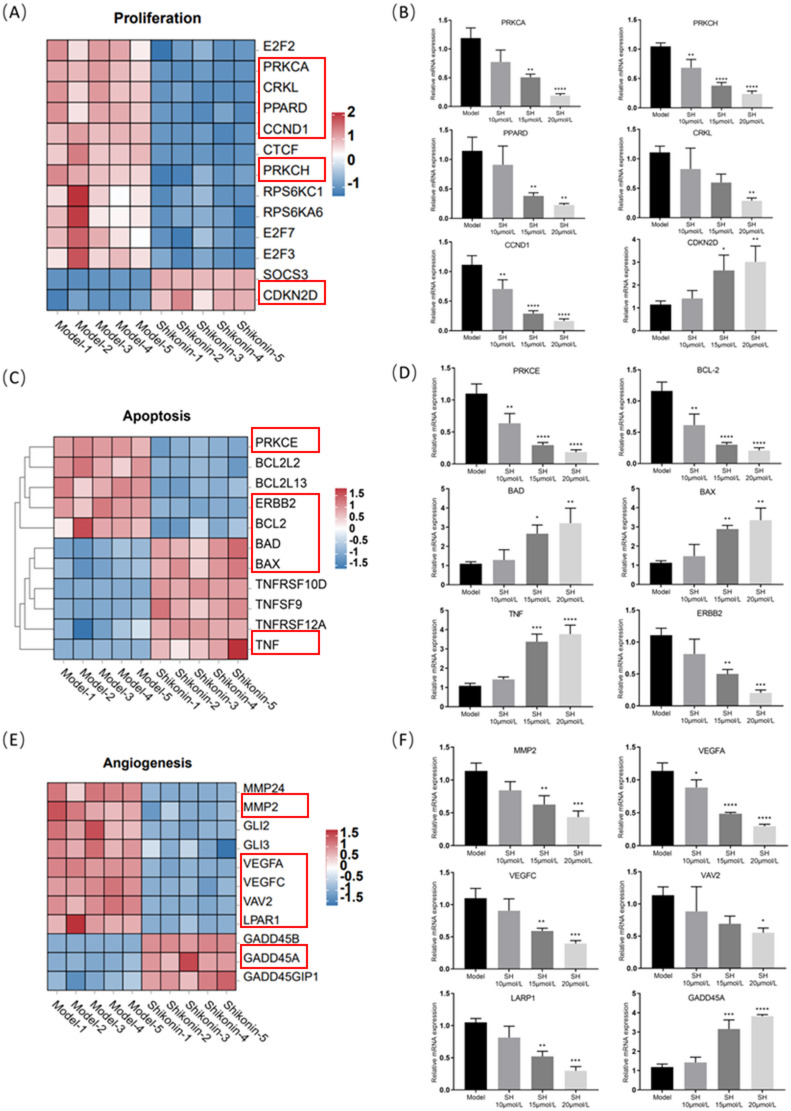
SH regulates the expression of DEGs related to tumor malignant phenotypes in A549 cells (TPM, n = 5 (independent cell cultures)). (**A**, **C**, **E**) Expression heatmaps for genes associated with proliferation, apoptosis, and angiogenesis derived from the RNA-seq dataset. (**B**) Relative mRNA levels of proliferation genes (PRKCA, PRKCH, PPARD, CRKL, CCND1, and CDKN2D). (**D**) Apoptosis gene mRNA expression levels (PRKCE, BCL-2, BAD, BAX, TNF, and ERBB2). (**E**) Expression levels of mRNA for genes related to angiogenesis (MMP-2, VEGFA, VEGFC, VAV2, LARP1, and GADD45A). TPM, transcripts per million. *p < 0.05; **p < 0.01; ***p < 0.001 and ****p < 0.0001, compared with Model group, one way ANOVA.

**Fig. 4:**
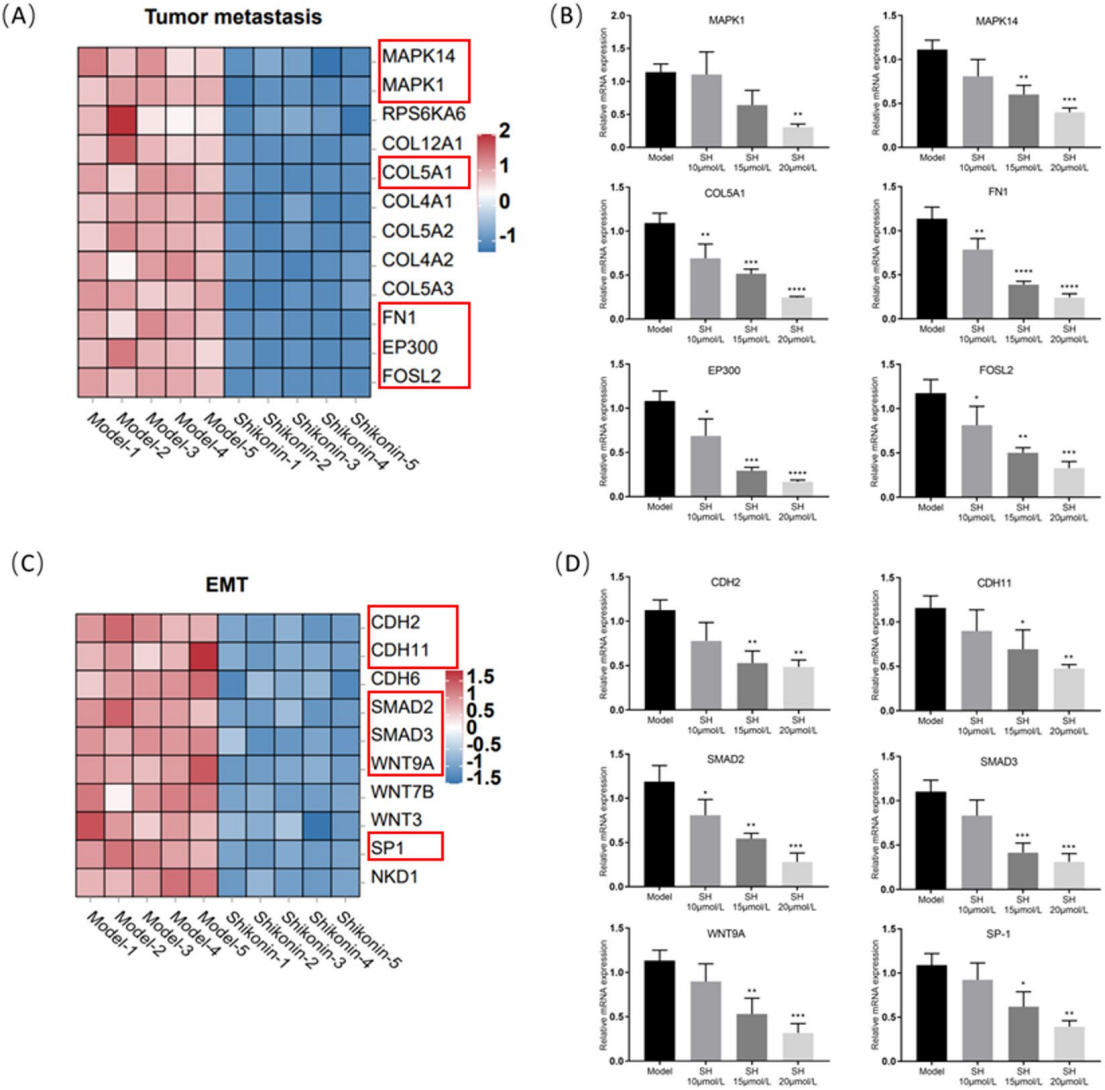
SH regulates the expression of DEGs related to tumor malignant phenotypes in A549 cells (TPM, n = 5 (independent cell cultures)). (**A**, **C**) Heatmaps of gene expression profiles related to tumor metastasis and EMT based on the RNA-seq data set. (**B**) Relative mRNA levels of tumor metastasis genes (MAPK1, MAPK14, COL5A1, FN1, EP300, and FOSL2). (**D**) Relative mRNA levels of EMT genes (CDH2, CDH11, SMAD2, SMAD3, WNT9A, and SP-1). TPM, transcripts per million. *p < 0.05; **p < 0.01; ***p < 0.001 and ****p < 0.0001, compared with Model group, one way ANOVA.

The heatmap based on the GSEA revealed SH’s regulation of DEGs related to tumor cell proliferation, apoptosis, and angiogenesis (Fig. 3A, C, E), as well as those associated with tumor metastasis and the epithelial-mesenchymal transition (EMT) (Fig. 4A, C). Subsequently, we validated the relevant DEGs via PCR. After the SH intervention, genes that promote proliferation, including PRKCA, PRKCH, PPARD, CRKL, and CCND1, were significantly downregulated after the SH intervention, while the expression of CDKN2D, a gene that inhibits proliferation, was significantly upregulated (Fig. 4B). Regarding genes related to apoptosis, the levels of the anti-apoptotic genes PRKCE, BCL-2, and ERBB2 were significantly downregulated after the SH intervention, while the levels of the pro-apoptotic genes BAD, BAX, and TNF were significantly upregulated (Fig. 3D). After the SH intervention, the levels of the tumor angiogenesis-related genes MMP-2, VEGFA, VEGFC, VAV2, and LARP1 were significantly downregulated when compared with the Model group, whereas the GADD45A level was markedly upregulated (Fig. 3F). The levels of genes associated with tumor metastasis, including MAPK1, MAPK14, COL5A1, FN1, EP300, and FOSL2, were significantly downregulated after SH intervention (Fig. 4B). Finally, the levels of genes involved in the tumor EMT, such as CDH2, CDH11, SMAD2, SMAD3, WNT9A, and SP-1, were significantly downregulated after the SH intervention (Fig. 4D). All the above results were consistent with the transcriptomic data.

### SH regulates the expression of tumor malignant phenotype markers in A549 cells

**Fig. 5:**
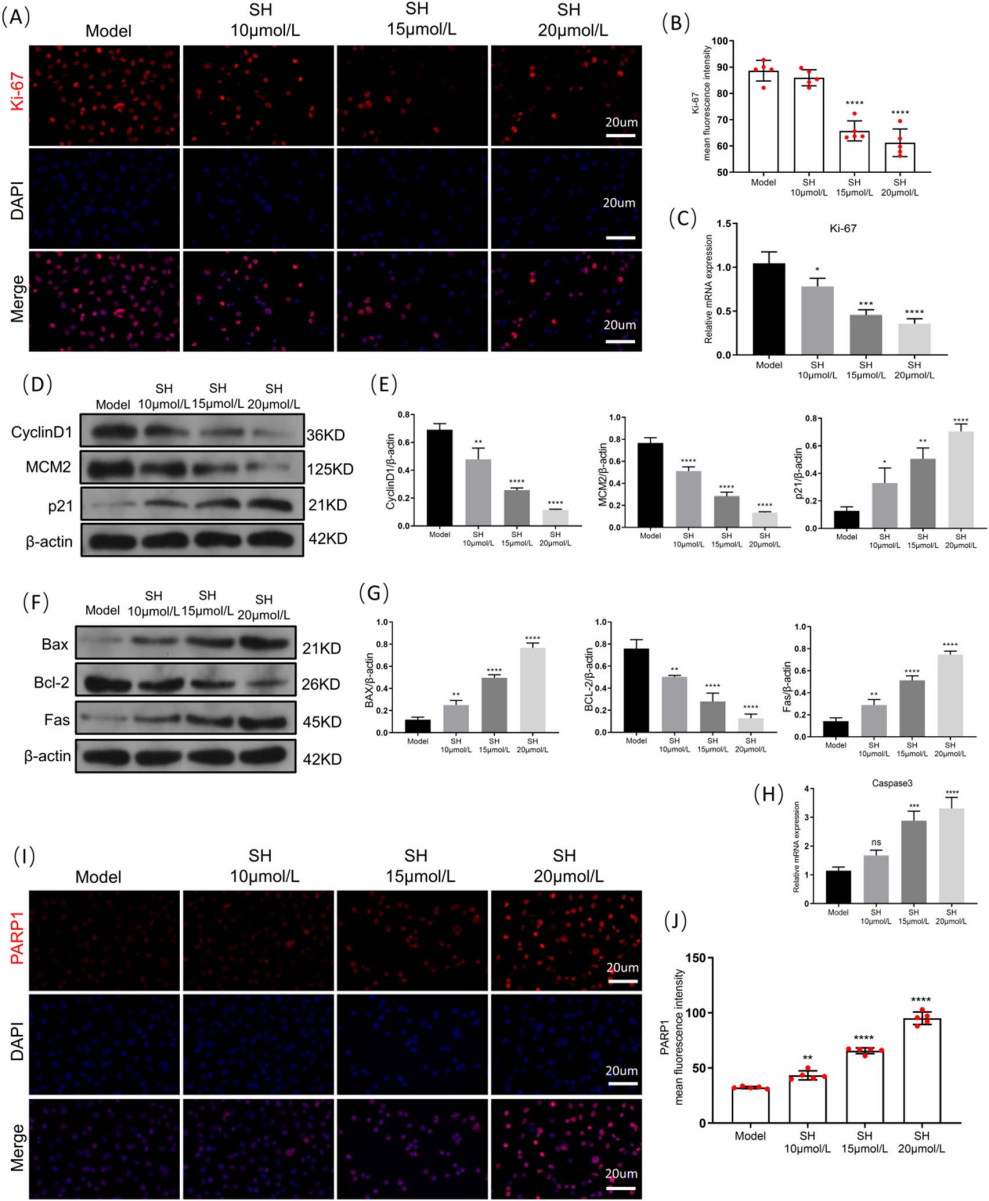
SH regulates the expression of tumor malignant phenotype markers in A549 cells (mean ± SD, n = 5 (independent cell cultures)). (**A**) Immunofluorescence staining of Ki‐67 in tumor cells (red) (scale bar: 20 μ m, × 400). (**B**) Quantification of intensity for ki-67 positive staining. (**C**) Relative mRNA levels of ki-67. (**D**) Representative Western blot analysis of CyclinD1, MCM2 and P21. (**E**) Quantitative analysis of the blot intensity of CyclinD1, MCM2 and P21 in D, n = 3. (**F**) Representative Western blot analysis of Bax, Bcl-2 and Fas. (**G**) Quantitative analysis of the blot intensity of Bax, Bcl-2 and Fas in F, n = 3. (**H**) Relative mRNA levels of Caspase3. (**I**) Representative images from immunofluorescence staining of PARP1 (red) (scale bar: 20 μ m, × 400). (**J**) Quantification of intensity for PARP1 positive staining. *p < 0.05; **p < 0.01; ***p < 0.001 and ****p < 0.0001, compared with Model group, one way ANOVA.

To further confirm the transcriptomic results, we used IF and PCR to detect the level of the tumor proliferation marker Ki-67 protein, thereby demonstrating a remarkable reduction in the number of Ki-67-positive cells and in Ki-67 mRNA expression during the SH intervention (Fig. 5A, B). The subsequent WB results indicated that SH decreased the levels of the proliferation-promoting proteins cyclin D1 and MCM2 and increased the expression of the proliferation-inhibiting factor p21 (Fig. 5C, D), further confirming the influence of SH on tumor cell proliferation. Next, we detected apoptosis-related proteins via WB, showing that SH elevated the levels of the pro-apoptotic proteins Bax and Fas, promoted caspase-3 mRNA expression, and reduced the level of the anti-apoptotic protein Bcl-2, while IF staining of PARP1, a core signaling molecule involved in tumor apoptosis inhibition (Fig. 5G, H), again confirmed SH’s promotion of tumor cell apoptosis.

### SH regulates the expression of angiogenesis, EMT, and metastasis-related proteins in A549 tumor cells

**Fig. 6:**
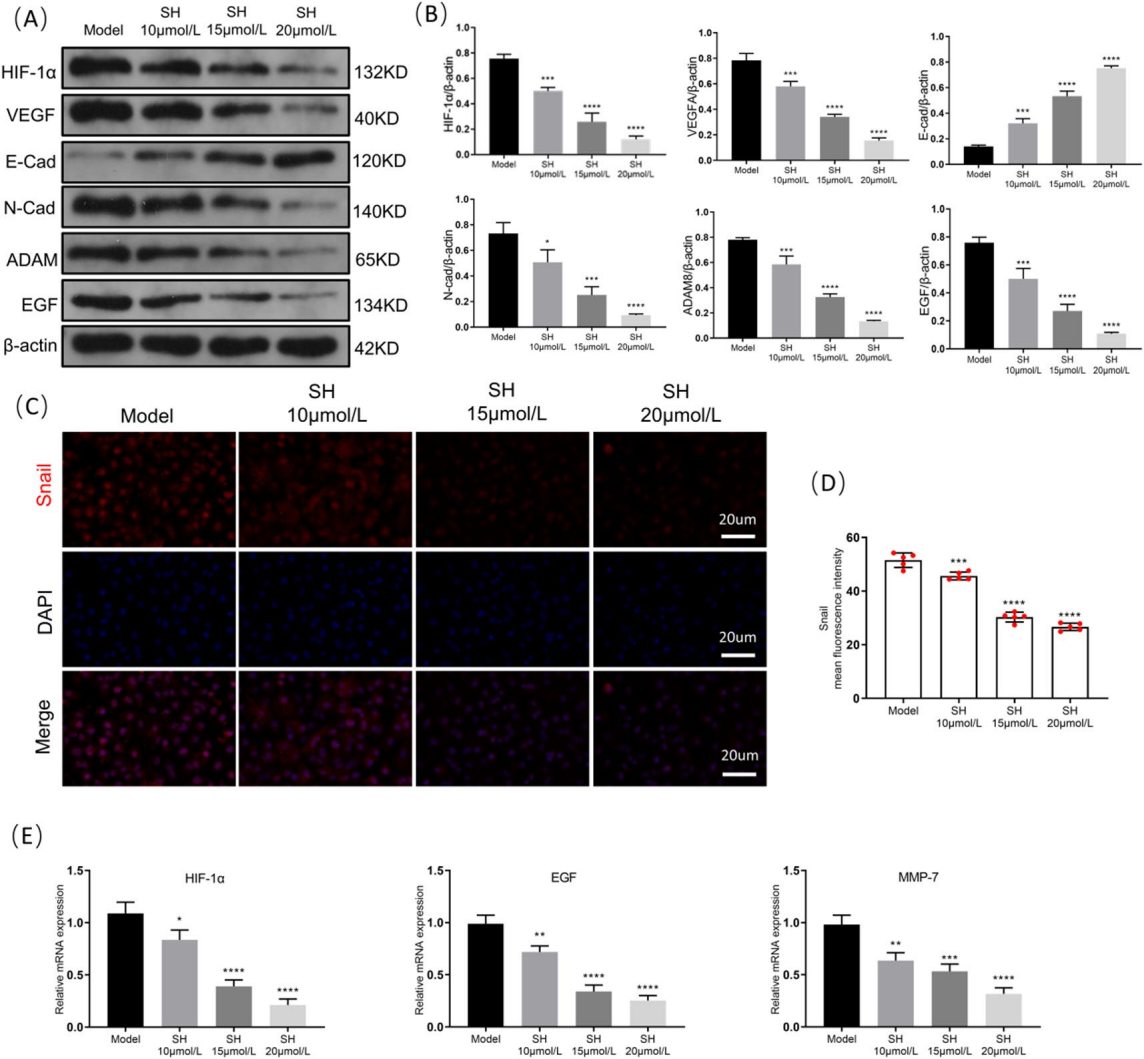
SH regulates the expression of angiogenesis, EMT, and metastasis-related proteins in A549 tumor cells (mean ± SD, n = 5 (independent cell cultures)). (**A**) Representative Western blot analysis of HIF-1α, VEGF, E-cad N-cad, Snail, ADAM and EGF. (**B**) Quantitative analysis of the blot intensity of HIF-1α, VEGF, E-cad N-cad, Snail, ADAM and EGF in A, n = 3. (**C**) Representative immunofluorescence images for Snail staining (red) (scale bar: 20 μ m, × 400). (**D**) Quantification of intensity for Snail positive staining. (**E**) Relative mRNA levels of HIF-1α, EGF and MMP-7. *p < 0.05; **p < 0.01; ***p < 0.001 and ****p < 0.0001, compared with Model group, one way ANOVA.

We assessed the changes in angiogenesis, EMT, and metastasis-related proteins of tumor cells following the SH intervention by means of WB and PCR. The WB findings revealed that SH reduced the levels of proteins such as HIF-1α, VEGF, N-cad, Snail, ADAM, and EGF and increased the protein expression of E-cad (Fig. 6A–D). The PCR findings demonstrated that SH reduced the mRNA levels of HIF-1α, EGF, and MMP-7 (Fig. 6E). These findings suggest that SH inhibits malignant phenotypes by regulating A549 cell proliferation, apoptosis, metastasis, angiogenesis, and EMT.

### SH inhibits lung cancer progression by regulating Hedgehog signaling pathway activation

**Fig. 7:**
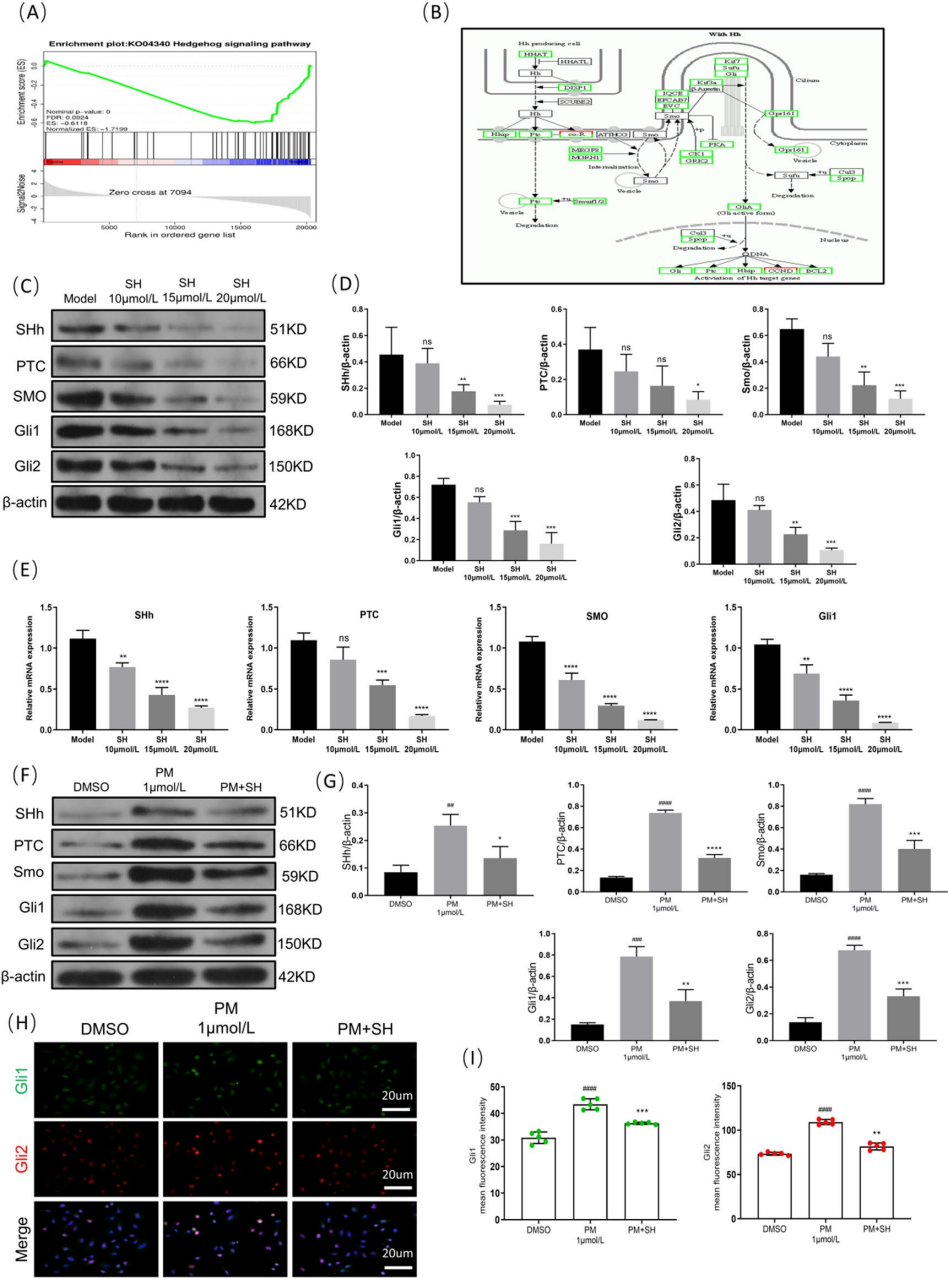
SH inhibits lung cancer progression by regulating Hedgehog signaling pathway activation (mean ± SD, n = 5 (independent cell cultures)). (**A**) Hedgehog signaling pathway, which is from the GSEA analysis of the gene profiling. (**B**) KEGG pathway analysis of Hedgehog signaling based on DEGs of Model group versus Shikonin group^5^, the green rectangle nodes represent down-regulated genes. (**C**) Representative Western blot analysis of SHh, PTC, SMO, Gli1 and Gli2. (**D**) Quantitative analysis of the blot intensity of SHh, PTC, SMO, Gli1 and Gli2 in C, n = 3. (**E**) Relative mRNA levels of SHh, PTC, SMO and Gli1. (**F**) Representative Western blot analysis of SHh, PTC, SMO, Gli1 and Gli2 after treatment with Hedgehog pathway agonist. (**G**) Quantitative analysis of the blot intensity of SHh, PTC, SMO, Gli1 and Gli2, n = 3. (**H**) Representative immunofluorescence images for Gli1 staining (green) and Gli2 staining (red), (scale bar: 20 μ m, × 400). (**I**) Quantification of intensity for Gli1 and Gli2 positive staining. *p < 0.05; **p < 0.01; ***p < 0.001 and ****p < 0.0001, compared with Model group. ^##^*p* < 0.01; ^###^*p* < 0.001 and ^####^*p* < 0.0001, compared with DMSO group. ^ns^*p* > 0.05, one way ANOVA.

The GSEA revealed significant enrichment of the Hedgehog pathway (Fig. 7A). Pathview was used to analyze the DEGs related to the Hedgehog pathway and the findings indicated that SH significantly downregulated the levels of genes associated with the Hedgehog pathway (Fig. 7B). When considered alongside the previously mentioned KEGG enrichment analysis results, these findings indicate that the Hedgehog signaling pathway may be a crucial factor in SH’s inhibition of primary lung cancer progression.

Prior studies have shown that targeting key points in the Hedgehog pathway, such as SHh, SMO, and GLI, can suppress lung cancer cell proliferation, enhance lung cancer cell apoptosis, and enhance drug sensitivity in drug-resistant lung cancer cells. Our study found that SH downregulated the protein and mRNA expression of SHh, PTC, SMO, Gli1, and Gli2 in the Hedgehog pathway (Fig. 7C–E), suggesting that SH might inhibit tumor progression via suppressing the Hedgehog pathway activation.

The subsequent validation with a Hedgehog pathway agonist revealed that the protein expression levels of SHh, PTC, SMO, Gli1, and Gli2 were markedly upregulated in the PM 1 μmol/L group when compared with the DMSO group (Fig. 7F, G). However, in the case of combined PM and SH intervention, the aforementioned findings were significantly reversed (Fig. 7F, G). Furthermore, after the PM treatment, the co-expression levels of the downstream proteins Gli1 and Gli2 in the Hedgehog pathway increased, while their co-expression levels were significantly decreased following the addition of SH (Fig. 7H, I). This further confirms SH’s role in inhibiting lung adenocarcinoma progression via the Hedgehog signaling pathway.

### SH improves the general state and tumor marker expression in lung cancer-bearing mice and transplanted tumors

**Fig. 8:**
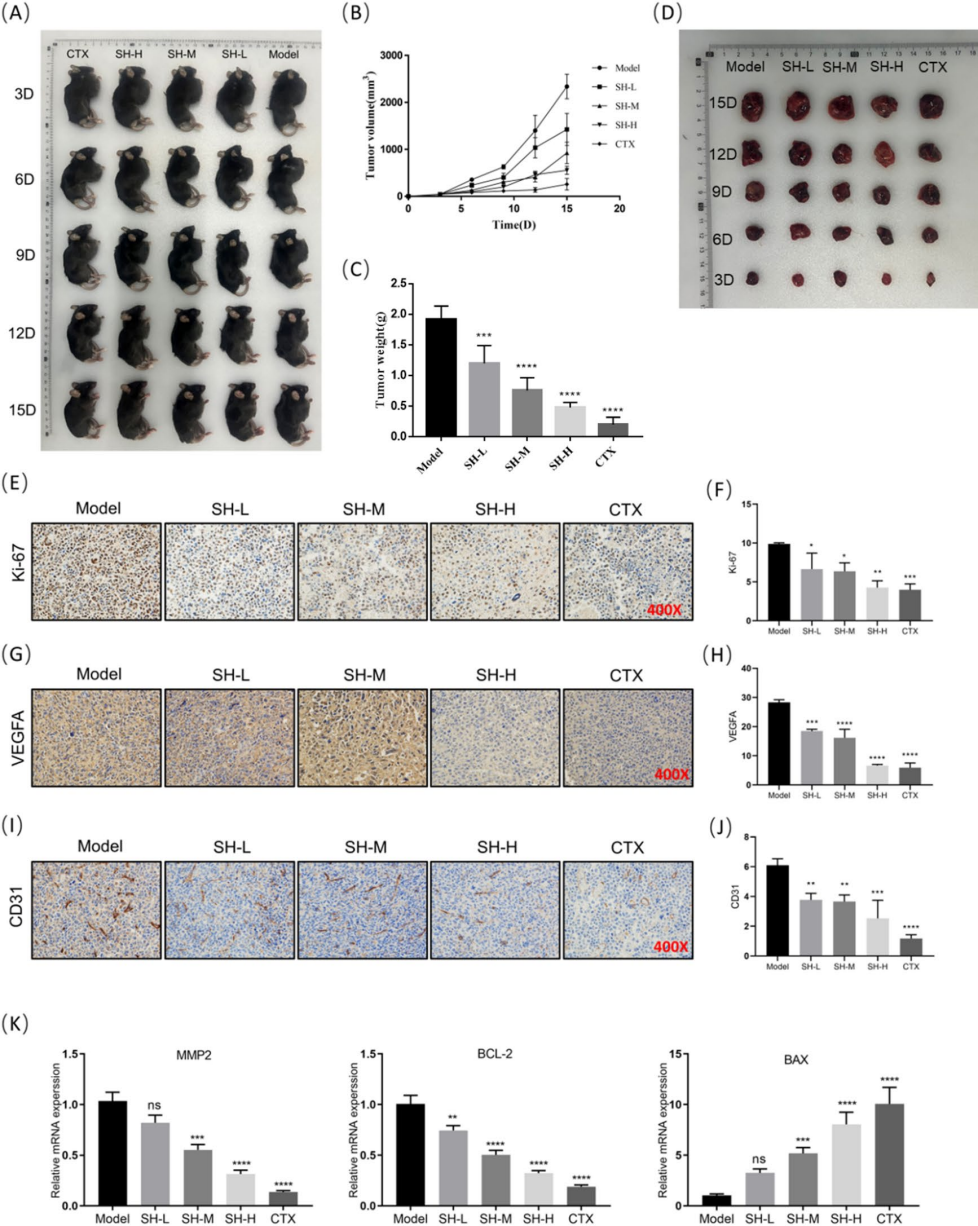
SH improves the general state and tumor marker expression in lung cancer-bearing mice and transplanted tumors (mean ± SD, n = 3 ~ 5(mice)). (**A**) Changes of subcutaneous tumors in different time periods, n = 3. (**B**) The tumor volume, n = 3. (**C**) The final tumor weight, n = 3. (**D**) The representative tumor images at the endpoint of study, n = 3. (**E**) The levels of ki-67 was evaluated by immunohistochemistry (scale bar: 20 μ m, × 400). (**F**) The intensity of ki-67 positive expression for IHC staining, n = 5. (**G**) The levels of VEGFA was evaluated by immunohistochemistry (scale bar: 20 μ m, × 400). (**H**) The intensity of VEGFA positive expression for IHC staining, n = 5. (**I**) The levels of CD31 was evaluated by immunohistochemistry (scale bar: 20 μ m, × 400). (**J**) The intensity of CD31 positive expression for IHC staining, n = 5. (**K**) Relative mRNA levels of MMP-2, BCL-2, BAX, n = 5. *p < 0.05; **p < 0.01; ***p < 0.001 and ****p < 0.0001, compared with Model group. ^ns^*p* > 0.05, one way ANOVA.

To further validate SH’s tumor suppression effect on lung adenocarcinoma in vivo, we established a xenograft tumor model with Lewis in C57BL/6 mice. After 15 days of SH administration, xenograft samples were collected and analyzed. We observed that the mouse xenograft tumor model was successfully constructed after 1 week of modeling (Tumor volume: 36–2662 mm^3^; weight: 0.1–2.2 g; diameter: 4–20 mm). The Model group exhibited larger tumor volumes and heavier weights when compared with the SH group, where the tumor volume and weight significantly decreased with an increasing SH intervention dose (Fig. 8A–D).

Additionally, the mice in the Model group displayed sluggish movement, lethargy, emaciation, and reduced food intake, indicating abnormal oxidative metabolism and impaired nutrient absorption and utilization due to tumor progression, which caused tumor-associated cachexia. Conversely, the mice treated with SH exhibited agile movement, good mental state, only slight emaciation, and normal food intake.

Finally, we detected the expression of proliferation, angiogenesis, and apoptosis markers in the mouse xenograft tissues and found that during the SH intervention, the protein levels of Ki-67, VEGFA, and CD31 in the xenograft tissue markedly reduced when compared with that in the Model group (Fig. 8E–J). Moreover, SH downregulated MMP-2 and Bcl-2 mRNA expression and promoted Bax mRNA expression (Fig. 8K). These results demonstrate that SH effectively suppresses the lung cancer xenograft growth in mice.

## Discussion

SH, as a naphthoquinone compound, is an active ingredient in the natural medicine *Lithospermum erythrorhizon* Siebold & Zucc and possesses anti-tumor effects. The present study investigated the role and related mechanisms of SH in inhibiting lung adenocarcinoma progression by means of transcriptomics and in vitro and in vivo experiments.

Tumor cells exhibit characteristics such as proliferation, metastasis, apoptosis resistance, angiogenesis, and EMT. While knowledge and understanding of the characteristics of tumors have been continually expanded and updated in recent years, these traits still fundamentally impact and reflect the progression of tumors. The most prominent feature of tumorigenesis is abnormal cell proliferation. Ki-67 is a nuclear antigen present in proliferating cells, and it is used to distinguish between tumor cells and normal cells in the growth phase, thereby being used as an index for tumor cell proliferation. Higher Ki-67 levels suggest a higher malignancy and a faster tumor cell proliferation rate^6^. Cyclin D1 and P21 are key proteins involved in the regulation of cell proliferation. More specifically, P21 inhibits cell cycle progression to play its anti-proliferation role by preventing cyclin D1 from binding to cyclin-dependent kinase (CDK), while cyclin D1 activates CDK to promote cell cycle progression, promoting both cell division and proliferation and leading to the occurrence and progression of various tumors^7,8^. MCM2 has been reported to have a nuclear localization function, which can guide multimers to accurately locate in the nucleus under normal conditions, thus participating in DNA replication. In the progression of tumors, MCM2 proteins, as specific indicators of cell proliferation, are expressed only in cells in the proliferation cycle^9^. Our study found that SH could inhibit Ki-67 and cyclin D1 protein expression in lung adenocarcinoma, promote p21 protein expression, and decrease the MCM2 protein expression levels, thus blocking tumor proliferation and inhibiting A549 cell proliferation activity.

Apoptosis is a key element in maintaining the cell balance in the body by eliminating unhealthy cells. Tumor cells avoid apoptosis signals via various pathways, maintaining an endless proliferative state^10^. Bax, a pro-apoptotic protein, is distributed in the cytoplasm of normal cells in a monomeric form. Upon activation of apoptosis signals, changes occur in the mitochondrial membrane potential and permeability, inducing the release of the apoptosis-inducing substance cytochrome C^11^. Bcl-2, a typical anti-apoptotic protein, forms heterodimers with Bax, thereby preventing Bax release and exhibiting anti-apoptotic effects^12^. Fas, an important member of the tumor necrosis factor receptor family, induces caspase-8 activation by binding to Fas L, thereby initiating cell apoptosis. The pro-apoptotic protein PARP1, as a cleavage substrate for the apoptosis executor caspase-3, causes cells to lose their DNA repair functions, leading them toward apoptosis^13^. Tumor cell apoptosis is inhibited. Our study found that after SH administration, apoptotic signal transduction was activated, increasing the Bax, Fas, PARP1, and caspase-3 protein expression while decreasing the Bcl-2 protein expression.

Angiogenesis provides sufficient blood supply and oxygen to tumor tissues, which is crucial for the “vigorous growth” of tumors. Tumors require abundant oxygen for cell proliferation, leading to hypoxic conditions in tumor tissues and often resulting in the overexpression of the hypoxia marker HIF-1α, which further stimulates tumor angiogenesis and participates in tumor cell energy metabolism and proliferation^14^. VEGF, a high-specific pro-vascular endothelial cell growth factor, plays a critical role in promoting angiogenesis around tumors^15^. This study found that SH administration could inhibit HIF-1α and VEGF protein expression, preventing tumor angiogenesis. With regard to the EMT, the Snail protein is a key transcription factor that promotes the tumor cell EMT, invasion, and migration. Overexpression of Snail can activate tumor suppressor genes and anti-apoptotic signals, enabling the endless proliferation of tumor cells^16^. N-cad and E-cad are functional transmembrane proteins involved in signal transduction. During the EMT process, Snail indirectly leads to abnormal expression of N-cad in tumors while suppressing E-cad expression, thereby enhancing the motility of tumor cells and significantly increasing the possibility of metastasis^17^. Our study found that after the SH intervention, the E-cad protein expression in the tumor cells increased, while the N-cad and Snail protein expression decreased. In terms of tumor metastasis, the ADAM protein family participates in extracellular matrix remodeling, altering cell adhesion ability and thus driving tumor invasiveness^18^. EGF has an anti-apoptotic effect on tumor cells, and high ECF expression can induce tumor cell adhesion and metastasis^19^. MMP-7 can promote normal cell proliferation and induce cancer cell aggregation, thereby participating in tumor occurrence and metastasis^20^. We observed a remarkable upregulation of the ADAM, EGF, and MMP-7 protein levels in lung adenocarcinoma, while after the SH intervention, these conditions were significantly reversed.

This study has confirmed the specific inhibitory effect of SH in relation to various malignant phenotypes of A549 cells. However, through what mechanism does it exert this effect? Our transcriptomics analysis revealed significant enrichment of the Hedgehog signaling pathway. Prior studies have shown that the Hedgehog signaling pathway is closely associated with tumorigenesis and malignant biological behaviors of tumors. Indeed, abnormal activation of this pathway can regulate the proliferation, angiogenesis, migration, invasion, and chemoresistance of tumor cells^21^. The Shh protein is a crucial component of the Hedgehog signaling family and can activate the downstream Gli1, which is a reliable marker of Shh pathway activity, regulating downstream oncogene transcription and thus playing a role in cancer cell survival, invasion, and self-renewal^22^. The Ptc protein, a 12-fold transmembrane protein, is capable of binding Hedgehog ligands and is a critical receptor protein in the Hedgehog signaling pathway, while Smo, a special sevenfold transmembrane protein, is a receptor necessary for the activation of Hedgehog signaling^23^. Gli2, an important transcription factor in the Hedgehog signaling pathway, is not only closely related to the growth of normal cells but is also abnormally activated in various tumor cells^24^. Our experiments revealed that SH regulated the Hedgehog signaling pathway in lung adenocarcinoma by downregulating the protein levels of the Hedgehog pathway nuclear transcription factor Gli1 and the Hedgehog pathway key factors SHh, Ptc, and Smo. In addition, compared with the DMSO group, the expression of key proteins in the Hedgehog signaling pathway was significantly upregulated in the PM intervention group, while co-culture of PM and SH inhibited the expression of these proteins.

Lastly, Lewis mouse transplant tumor experiments showed that SH inhibited tumor growth, reduced the protein expression of Ki-67, VEGFA and CD31 in transplanted tumor tissues, and the mRNA expression of MMP-2 and Bcl-2, while promoting the expression of Bax mRNA. These results indicate that SH can effectively inhibit tumor proliferation, angiogenesis, and EMT and promote tumor cell apoptosis in lung adenocarcinoma in vivo.

Our research has found that shikonin may inhibit the malignant phenotype of lung adenocarcinoma by regulating the Hedaege signaling pathway. New research has found that the transmembrane protein 16A (TMEM16A) chloride channel promotes lung cancer cell growth and invasion through an epidermal growth factor receptor (EGFR)/MAPK-dependent signaling pathway^25^. Notably, studies have shown that shikonin can inhibit TMEM16A chloride channel activity in mice, thereby reducing intestinal motility and treating secretory diarrhea^26^. However, whether it can inhibit the TMEM16A chloride channel in lung adenocarcinoma cells requires further investigation in the future.However, it must be borne in mind that this study is limited to SH’s anti-tumor effects on lung adenocarcinoma, lacks animal safety testing, is slightly limited in terms of the in vitro experimental cell types, and lacks exploration of the action sites of SH regulating Hedgehog pathway. Shikonin has potential for future medicinal applications, but it is still in the animal testing stage and may be significantly different from current NSCLC/lung adenocarcinoma treatment strategies^27^. Future research should delve into SH’s regulation of the Hedgehog pathway through methods such as molecular docking and surface plasmon resonance and expand understanding of its effects on other subtypes of lung cancer.

## Conclusion

This study comprehensively investigated the role of SH in inhibiting the progression of NSCLC, particularly lung adenocarcinoma, through transcriptomic analyses combined with in vitro and in vivo experiments. SH inhibited lung cancer A549 cell proliferation, angiogenesis, apoptosis inhibition, metastasis, and EMT and regulated the expression of multiple mRNAs related to various malignant phenotypes, possibly by suppressing Hedgehog pathway activation.

## Supplementary Information

Below is the link to the electronic supplementary material.

Supplementary Material 1

Supplementary Material 2

## Data Availability

The data used to support the findings of this study are available from the corresponding author upon reasonable request.
