# Supplementary material for: Shikonin inhibits multiple tumor malignant phenotypes and is associated with Hedgehog pathway downregulation in lung adenocarcinoma
Source: Sci Rep. 2025 Dec 24;15:44516. doi: 10.1038/s41598-025-28080-9 (PMC12738551; doi:10.1038/s41598-025-28080-9)
Supplement: Supplementary file 1 — Supplementary Material 1 [file 41598_2025_28080_MOESM1_ESM.docx]

**Supplementary Table 1 Primers for RT-PCR analysis**

| Gene | Primer | Sequence (5'-3') | PCR Products |
| --- | --- | --- | --- |
| β-actin | Forward | CCCTGGAGAAGAGCTACGAG | 180bp |
|  | Reverse | CGTACAGGTCTTTGCGGATG |  |
| PRKCA | Forward | AAACGGGCTTTCAGATCCTT | 193bp |
|  | Reverse | CCTTGTTGTTCGATCCCAGT |  |
| PRKCH | Forward | CAGAGGTTCGGGATCAACAT | 236bp |
|  | Reverse | ATATTTCCGGGTTGGAGACC |  |
| PPARD | Forward | ACTGAGTTCGCCAAGAGCAT | 239bp |
|  | Reverse | GCGTTGAACTTGACAGCAAA |  |
| CRKL | Forward | ATCTGTCTCAGCACCCAACC | 157bp |
|  | Reverse | GGCACTCCACCACTGTTCTT |  |
| CCND1 | Forward | CGGACTACAGGGGAGTTTTG | 273bp |
|  | Reverse | AGGAGGTTGGCATCGGGGT |  |
| CDKN2D | Forward | GTCATGATGTTTGGCAGCACG | 223bp |
|  | Reverse | GTCATGATGTTTGGCAGCAC |  |
| PRKCE | Forward | GATGCAGAAGGTCACTGCAA | 249bp |
|  | Reverse | GTCGTCATGGAGGATGGACT |  |
| BCL2 | Forward | AGGATTGTGGCCTTCTTTGA | 169bp |
|  | Reverse | ACAGTTCCACAAAGGCATCC |  |
| BAD | Forward | CCGAGTGAGCAGGAAGACTC | 205bp |
|  | Reverse | GGTAGGAGCTGTGGCGACT |  |
| BAX | Forward | TCTGACGGCAACTTCAACTG | 217bp |
|  | Reverse | ACAGGGACATCAGTCGCTTC |  |
| TNF | Forward | TCAGAGGGCCTGTACCTCAT | 220bp |
|  | Reverse | GGAAGACCCCTCCCAGATAG |  |
| ERBB2 | Forward | CGCTTTTGGCACAGTCTACA | 249bp |
|  | Reverse | TCCCGGACATGGTCTAAGAG |  |
| MMP2 | Forward | ACCACAGCCAACTACGATGA | 202bp |
|  | Reverse | GCTCCTGAATGCCCTTGATG |  |
| VEGFA | Forward | GGAGGAGGGCAGAATCATCA | 247bp |
|  | Reverse | CTTGGTGAGGTTTGATCCGC |  |
| VEGFC | Forward | ACCAAACAAGGAGCTGGATG | 237bp |
|  | Reverse | CAGGCACATTTTCCAGGATT |  |
| VAV2 | Forward | TCAAGGTGCATCACAGCTTC | 208bp |
|  | Reverse | TTCAGTGTGCACTCCTCGAC |  |
| LARP1 | Forward | AACTACCGCAACACCAGGAC | 250bp |
|  | Reverse | CTGTAGGCGTCCCTTCTGAG |  |
| GADD45A | Forward | CTGGAGAGCAGAAGACCGAA | 201bp |
|  | Reverse | CAGCGTCGGTCTCCAAGA |  |
| MAPK1 | Forward | ACTAACGTTCTGCACCGTGAC | 226bp |
|  | Reverse | GAGAATGCAGCCTACAGACCA |  |
| MAPK14 | Forward | GCCAAGCCATGAGGCAAGAAACTAT | 126bp |
|  | Reverse | TCCAATACAAGCATCTTCTCCAGCA |  |
| EP300 | Forward | CAGATTGATCCCAGCTCCAT | 214bp |
|  | Reverse | AAAGAAGACTCGGCGTTTGA |  |
| FOSL2 | Forward | TCTGTCATCAAGCCCATCAG | 237bp |
|  | Reverse | AGTGGGGGAGTTCAAGGAGT |  |
| L5A1 | Forward | GGCATCGGGGACTATGACTA | 216bp |
|  | Reverse | CTGTAGGCGTCCCTTCTGAG |  |
| FN1 | Forward | TCCCCAACTGGTAACCCTTC | 250bp |
|  | Reverse | CGGGTATGGTCTTGGCCTAT |  |
| CDH2 | Forward | AGGGGACCTTTTCCTCAAGA | 190bp |
|  | Reverse | TCAAATGAAACCGGGCTATC |  |
| CDH11 | Forward | CATCAGAACAGCCCTACCCA | 160bp |
|  | Reverse | CGGAAACTTTGGTGGGTTGT |  |
| SMAD2 | Forward | CGTCTCCAGGTATCCCATCG | 223bp |
|  | Reverse | TTAGGATCTCGGTGTGTCGG |  |
| SMAD3 | Forward | ACGACTACAGCCATTCCATCCC | 128bp |
|  | Reverse | CATCTGGTGGTCACTGGTTTCTC |  |
| WNT9A | Forward | GCAAGCATCTGAAGCACAAG | 179bp |
|  | Reverse | GCAGAAGCTAGGCGAGTCAT |  |
| SP1 | Forward | AGGGGTCTGATGCTCTGAACA | 209bp |
|  | Reverse | CTGGGAGATGGCTTGAGTTGTA |  |
| Ki-67 | Forward | AAGCCCTCCAGCTCCTAGTC | 222bp |
|  | Reverse | TCCGAAGCACCACTTCTTCT |  |
| caspase 3 | Forward | ACTGGACTGTGGCATTGAGA | 162bp |
|  | Reverse | GCACAAAGCGACTGGATGAA |  |
| HIF-1α | Forward | GTGGCGAAGATGGTCAAGTC | 116bp |
|  | Reverse | GGAGTGCCCTTGTTGAGGTGTT |  |
| EGF | Forward | TCACCTCAGGGAAGATGACC | 193bp |
|  | Reverse | CAGTTCCCACCACTTCAGGT |  |
| MMP-7 | Forward | AAAGAGATCCCCCTGCATTT | 162bp |
|  | Reverse | GTGAGCATCTCCTCCGAGAC |  |
| sHh | Forward | CTTCTCCTCCTGATGCTTTTGC | 318bp |
|  | Reverse | CTCCTTGCTGTGGTTGTGATTT |  |
| PTC | Forward | ACAAACTCCTGGTGCAAACC | 207bp |
|  | Reverse | CTTTGTCGTGGACCCATTCT |  |
| SMO | Forward | TCACTCCCCTTTGTCCTCAC | 250bp |
|  | Reverse | TGGTCTCGTTGATCTTGCTG |  |
| Gli1 | Forward | GTGCAAGTCAAGCCAGAACA | 162bp |
|  | Reverse | ATAGGGGCCTGACTGGAGAT |  |
