# Supplementary material for: Shikonin inhibits multiple tumor malignant phenotypes and is associated with Hedgehog pathway downregulation in lung adenocarcinoma
Source: Sci Rep. 2025 Dec 24;15:44516. doi: 10.1038/s41598-025-28080-9 (PMC12738551; doi:10.1038/s41598-025-28080-9)
Supplement: Supplementary file 2 — Supplementary Material 2 [file 41598_2025_28080_MOESM2_ESM.pdf]

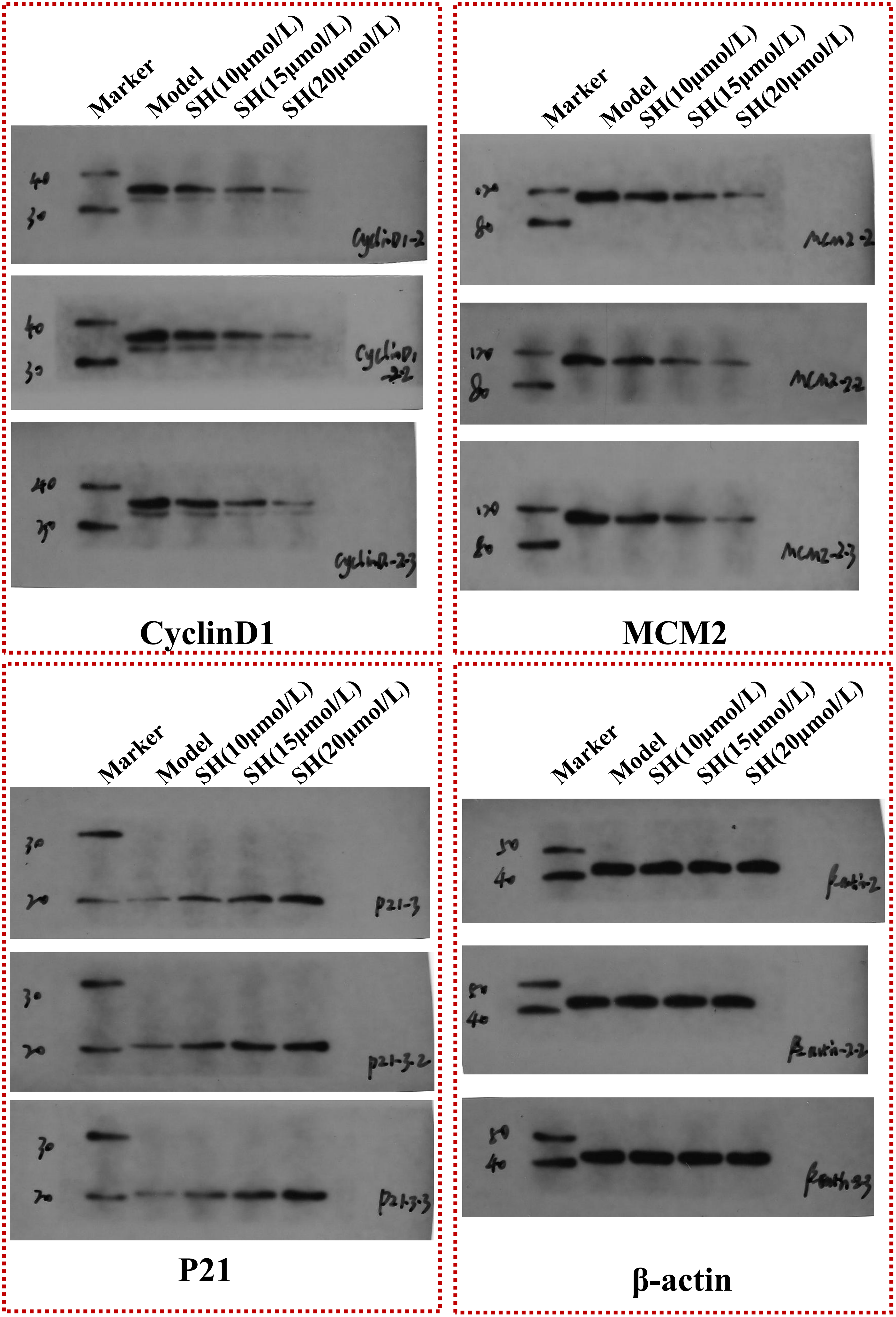
Marker
Model
SH(10μmol/L)
SH(15μmol/L)
SH(20μmol/L)
CyclinD1
MCM2
P21
β-actin

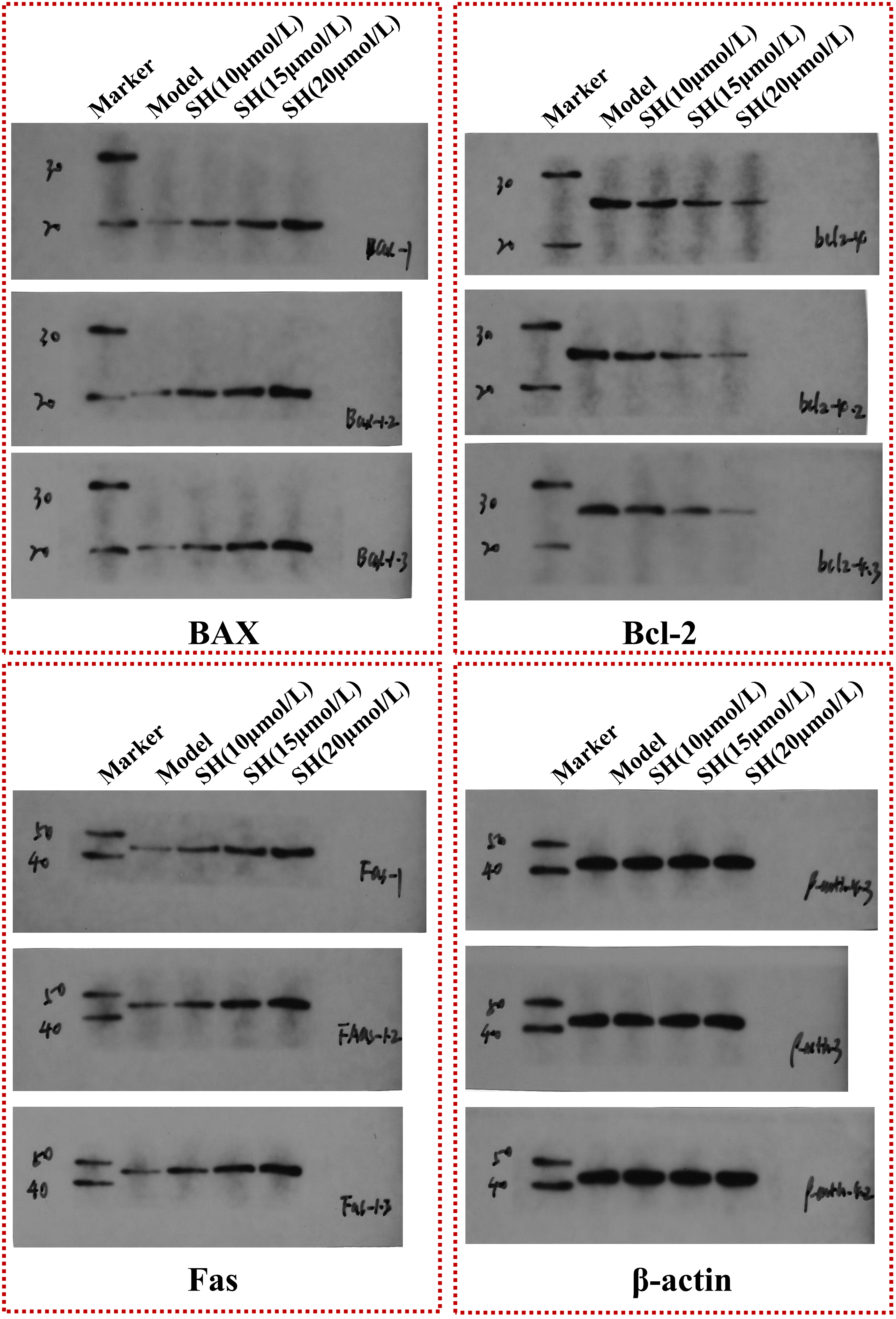
Marker
Model
SH(10μmol/L)
SH(15μmol/L)
SH(20μmol/L)
BAX
Bcl-2
Fas
β-actin

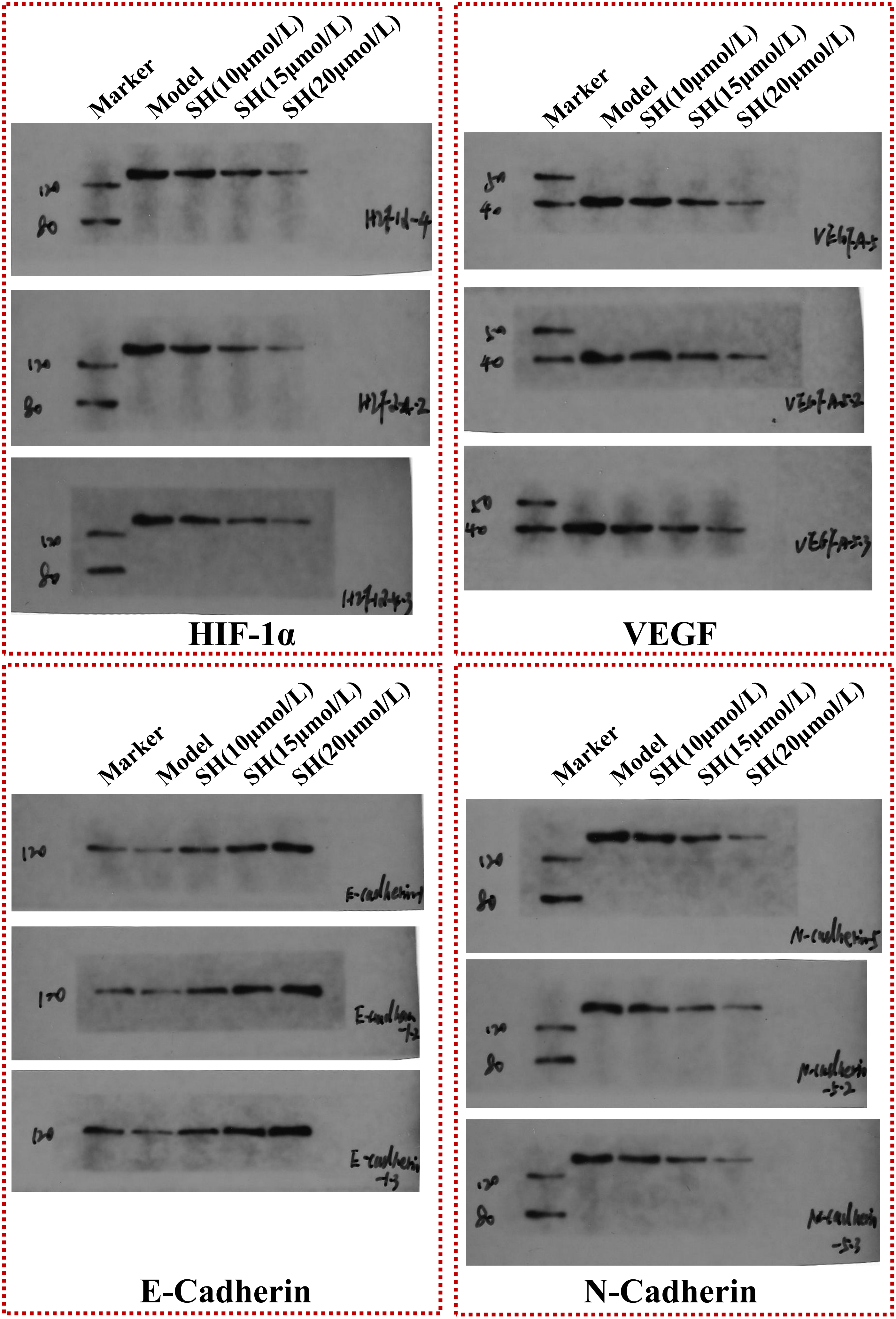
Marker
Model
SH(10μmol/L)
SH(15μmol/L)
SH(20μmol/L)
HIF-1α
VEGF
E-Cadherin
N-Cadherin

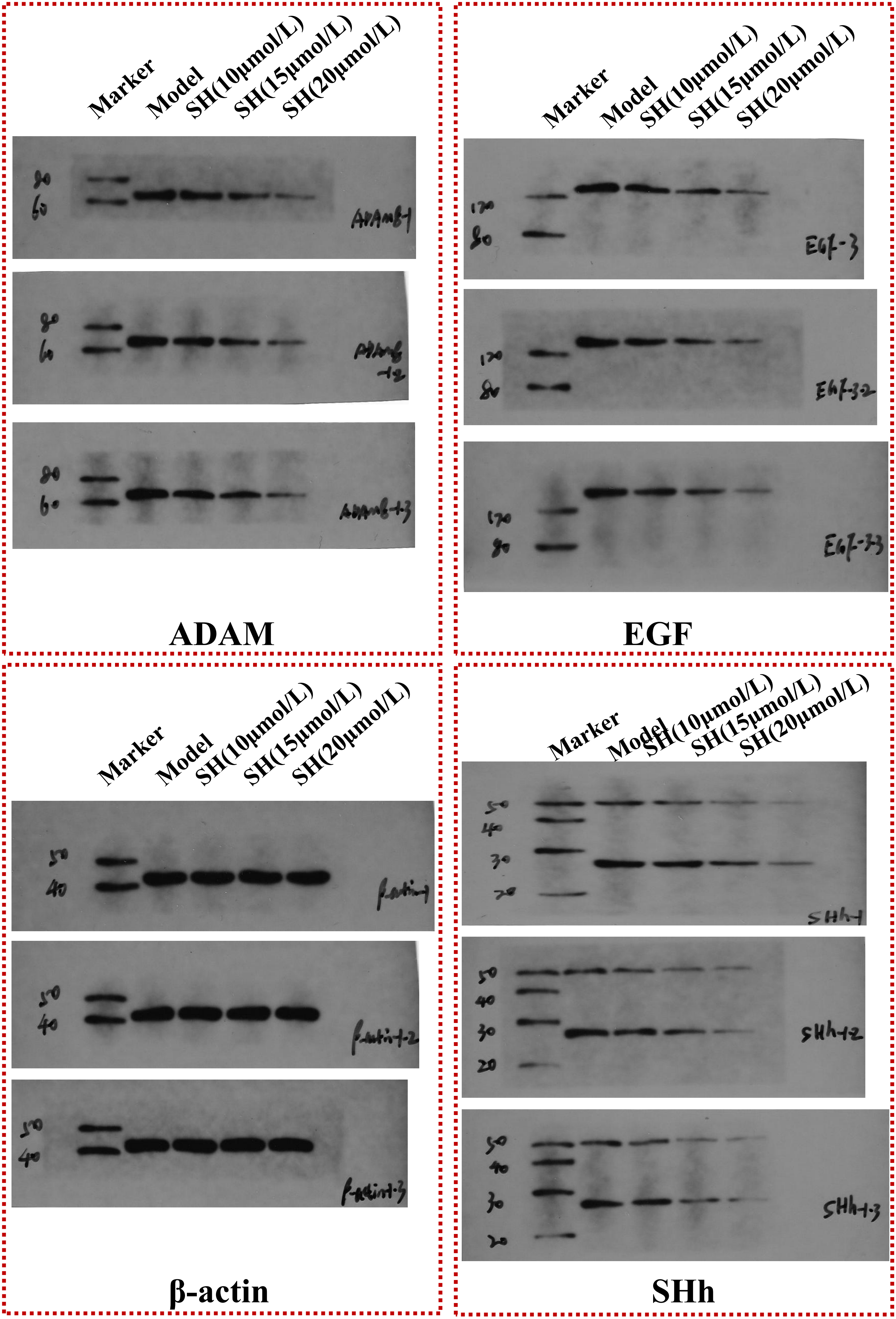
Marker
Model
SH(10μmol/L)
SH(15μmol/L)
SH(20μmol/L)
ADAM
EGF
β-actin
SHh

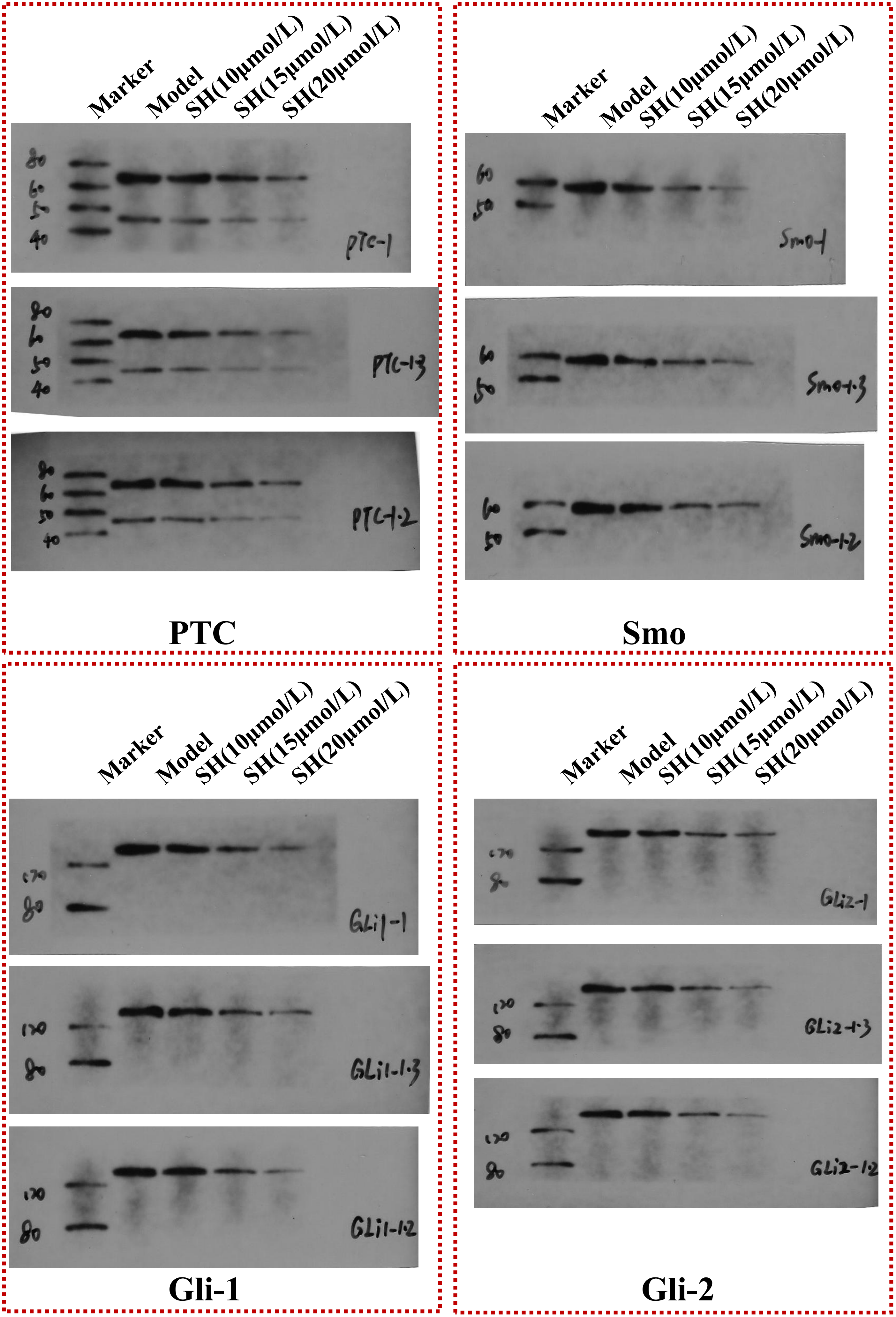
Marker
Model
SH(10μmol/L)
SH(15μmol/L)
SH(20μmol/L)
PTC
Smo
Gli-1
Gli-2

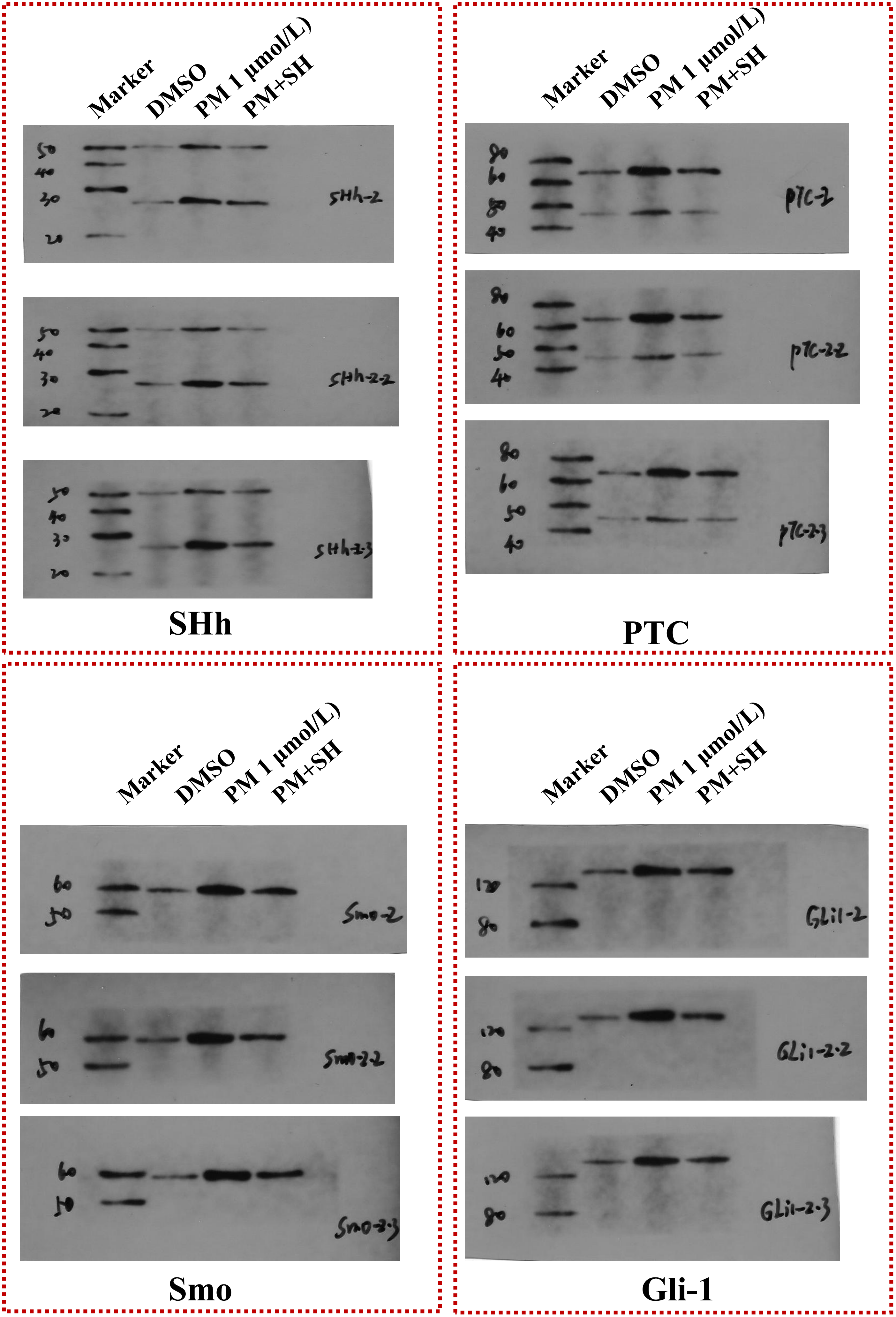
Marker
DMSO
PM 1 μmol/L)
PM+SH
SHh
PTC
Smo
Gli-1

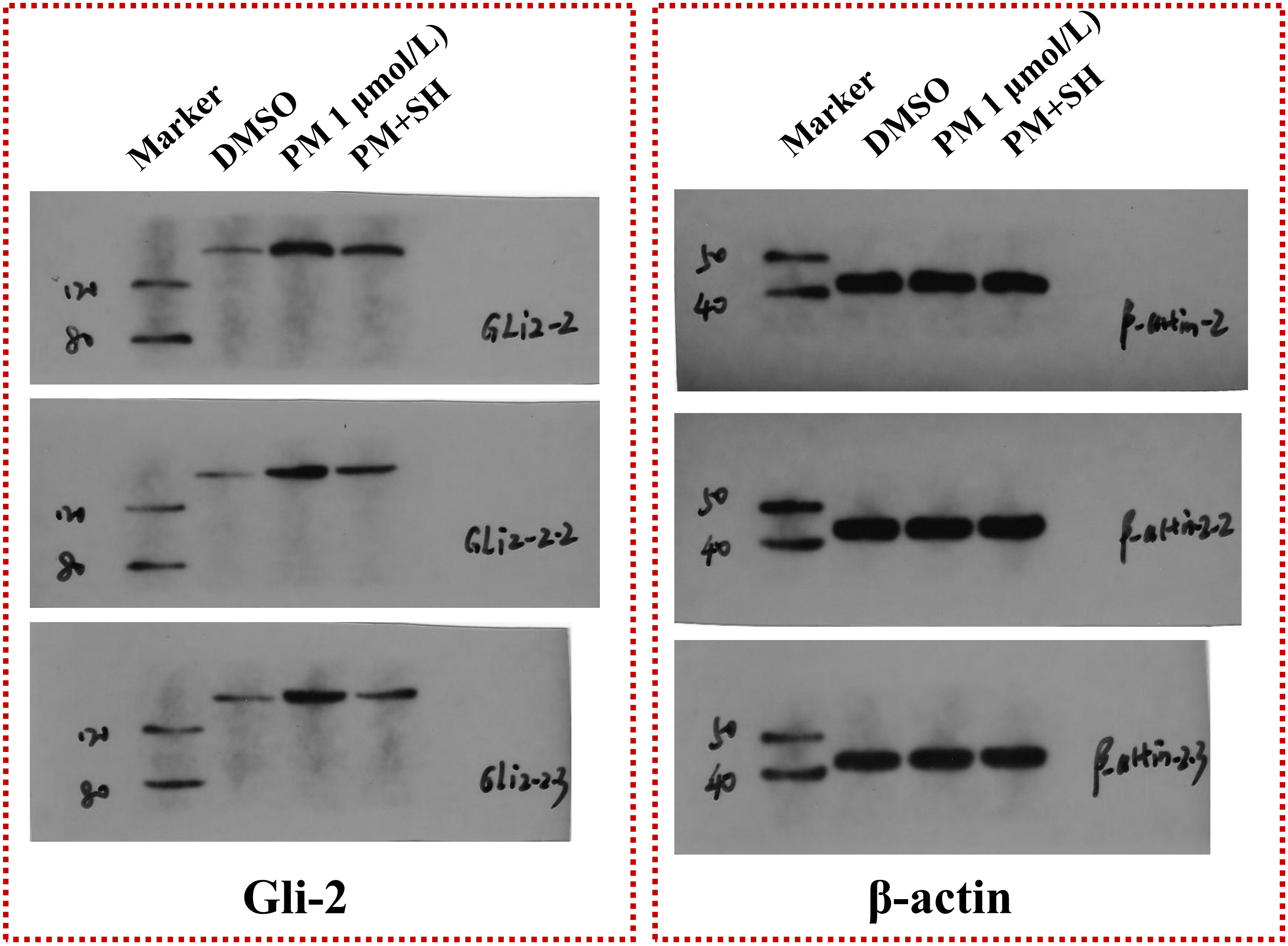
Marker
DMSO
PM 1 μmol/L)
PM+SH
120
80
Gli-2
Marker
DMSO
PM 1 μmol/L)
PM+SH
50
40
β-actin
